# Analysis of Mutant SOD1 Electrophoretic Mobility by Blue Native Gel Electrophoresis; Evidence for Soluble Multimeric Assemblies

**DOI:** 10.1371/journal.pone.0104583

**Published:** 2014-08-14

**Authors:** Hilda H. Brown, David R. Borchelt

**Affiliations:** Department of Neuroscience, Center for Translational Research in Neurodegenerative Disease, SantaFe HealthCare Alzheimer's Disease Research Center, McKnight Brain Institute, University of Florida College of Medicine, Gainesville, Florida, United States of America; Children's Hospital of Pittsburgh, University of Pittsburgh Medical Center, United States of America

## Abstract

Mutations in superoxide dismutase 1 (SOD1) cause familial forms of amyotrophic lateral sclerosis (fALS). Disease causing mutations have diverse consequences on the activity and half-life of the protein, ranging from complete inactivity and short half-life to full activity and long-half-life. Uniformly, disease causing mutations induce the protein to misfold and aggregate and such aggregation tendencies are readily visualized by over-expression of the proteins in cultured cells. In the present study we have investigated the potential of using immunoblotting of proteins separated by Blue-Native gel electrophoresis (BNGE) as a means to identify soluble multimeric forms of mutant protein. We find that over-expressed wild-type human SOD1 (hSOD1) is generally not prone to form soluble high molecular weight entities that can be separated by BNGE. For ALS mutant SOD1, we observe that for all mutants examined (A4V, G37R, G85R, G93A, and L126Z), immunoblots of BN-gels separating protein solubilized by digitonin demonstrated varied amounts of high molecular weight immunoreactive entities. These entities lacked reactivity to ubiquitin and were partially dissociated by reducing agents. With the exception of the G93A mutant, these entities were not reactive to the C4F6 conformational antibody. Collectively, these data demonstrate that BNGE can be used to assess the formation of soluble multimeric assemblies of mutant SOD1.

## Introduction

Amyotrophic Lateral Sclerosis (ALS) is primarily characterized by loss of upper and lower motor neurons and usually arises by an unknown etiology (sporadic ALS or sALS). Between 10 and 20% of ALS cases can be linked to defined genetic causes (familial ALS or fALS), and of these inherited genetic mutations, approximately 20% are found in the Cu-Zn superoxide dismutase (*sod1*) gene [1]. Over 150 mutations in *sod1* have been linked to fALS {http://alsod.iop.kcl.ac.uk/default.aspx}. The vast majority of these inherited mutations are missense point mutations, but there are also examples of mutations that result in early termination of translation, yielding truncated proteins that lack residues encoded in the 5^th^ and last coding exon. There are also rare reports of frame-shift and early terminations that would produce mRNAs that would be predicted to be degraded by nonsense mediated decay pathways {http://alsod.iop.kcl.ac.uk/default.aspx}. However, whether these mutations are causative of disease in these individuals is uncertain.

The *sod1* gene encodes a protein of the same name, SOD1, which is a relatively small protein of 153 amino acids that contains eight β-strands folded into a flattened barrel-like structure [2], [3]. When isolated from tissues and cells, the wild-type (WT) protein is normally found as a homodimer composed of two subunits in which each subunit binds one atom of copper and one atom of zinc [4]. The binding of metals, along with the formation of an intramolecular disulfide bond between cysteine 57 and cysteine 146 is thought to provide high thermal stability to the protein [5].

The mechanisms by which mutations in SOD1 cause motor neuron degeneration have yet to be clearly delineated (for review see [6]). The effects of fALS mutations on the normal enzyme activity and protein turnover vary greatly [7]–[11]. While some mutants are rapidly degraded or inactive, others retain high levels of activity and relatively long half-lives [7]–[16]. Various studies have demonstrated that fALS mutations in SOD1 heighten susceptibility of the protein to misfold and aggregate into large detergent-insoluble structures [17]–[20]. Although the role of mutant protein aggregates in acquired neurotoxicity has not been defined, mutations that show a high propensity to aggregate are generally associated with forms of the disease that show more rapid rates of progression [20].

In addition to large, sedimentable aggregates, smaller oligomeric assemblies of mutant SOD1 have been detected by various forms of size exclusion chromatography [21]–[24]. In the present study, we have assessed the feasibility of detecting oligomeric forms of mutant SOD1 by immunoblotting of blue-native polyacrylamide gels, which were initially developed as a means to examine multimeric protein complexes [25]. Blue-native polyacrylamide gel electrophoresis (BNGE) utilizes the binding of Coomassie blue dye to proteins to impart net negative charge so that the protein will migrate into the polyacrylamide gel. The use of Coomassie blue replaces ionic detergents such as sodium dodecyl sulfate (SDS) as the source of negative charge in gel electrophoresis and allows for detergent labile structures to remain intact [25]. Native gel electrophoresis for SOD1 zymographs have also been used to study SOD1 [7], but our experience with immunoblotting of these types of gels has been very mixed and thus we here investigate the utility of BNGE in detecting soluble forms of SOD1 that may be assembled into multimeric or multi-protein complexes. The model system used to investigate this question involved transient over-expression of the protein in cultured human 293 cells, a system we have used extensively to study mutant SOD1 aggregation [20], [26]–[31]. In this system, the mutant SOD1 is greatly over-expressed in a subset of cells and in such setting the formation of homo-multimeric structures (e.g. oligomeric assemblies of SOD1) would be favored over hetero-multimeric complexes (complexes of SOD1 with other proteins) because the molar ratio of SOD1 to other binding partners is skewed. We report here that we can detect, to varying degrees, entities by BNGE that show electrophoretic mobility consistent with oligomeric structures.

## Methods

### Reagents

Purified WT-hSOD1 protein as isolated from yeast and WT-hSOD1-apo (lacking Cu) was provided by Dr. P. John Hart (University of Texas Health Sciences Center San Antonio). Antibodies to SOD1 included the following, a rabbit polyclonal peptide antiserum raised against the human peptide sequence 24-36 (hSOD1) that specifically recognizes human SOD1 [32], a monoclonal antibody raised against the apo-form of human SOD1-G93A [33] (termed C4F6, Medimabs, Montreal, Canada), and a rabbit polyclonal antiserum raised against a peptide representing amino acids 145-151 in human SOD1 (termed SEDI, graciously provided by Dr. Janice Robertson, University of Toronto [34]). The antibody to ubiquitin (Ubi-1) was purchased from EnCor Biotechnology (Gainesville, FL, cat#MCA-Ubi-1). All cell culture reagents were purchased from HyClone (Logan, Utah) and all other chemicals were purchased from other vendors as noted.

### Cell transfection

The cDNA vectors used to express WT and mutant SOD1 and the methods used in transfection of these plasmids into HEK 293FT cells have been previously described. The vectors encoding SOD1-A4V, SOD1-G37R, and SOD1-G85R were described in [7]. The vector encoding SOD1-C6F/C57S/C111Y/C146R (SOD1-FSYR) was described in [27]. The vector encoding SOD1-G93A was described in [20] and the vector encoding SOD1-L126Z was described in [13]. All SOD1 expression plasmids were based on the pEF.BOS vector [35] with 4 µg of these plasmids being transiently transfected into HEK293FT cells (Invitrogen/Life Technologies, Grand Island, NY, USA) using Lipofectamine 2000 (Invitrogen/Life Technologies).

### BNGE and Immunoblotting

**Figure 1:**
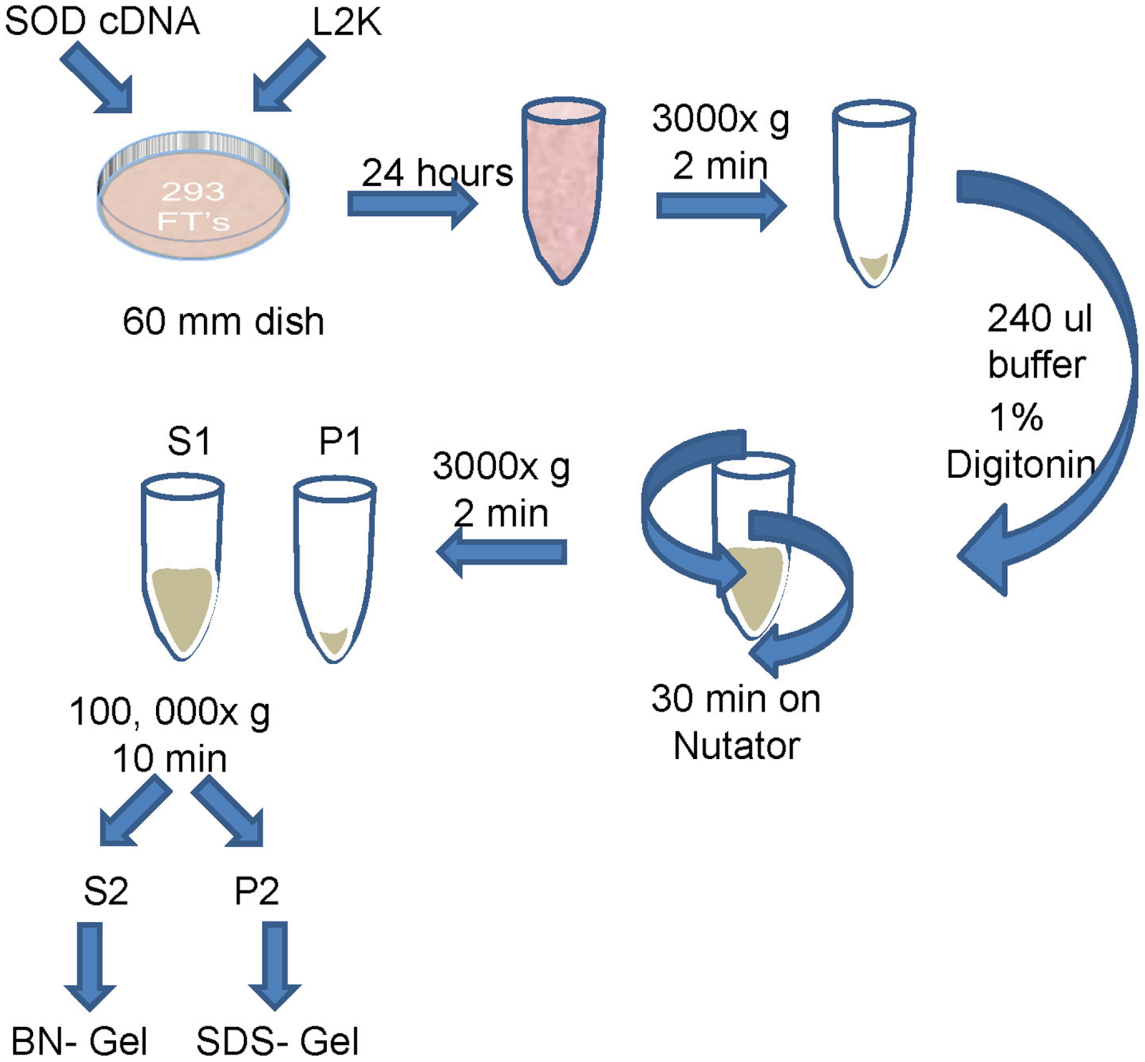
Schematic of the methodology for sample preparation for BN-gels. A detailed description of the methods is provided in the Methods Section.

The methods used in BNGE were essentially that provided by the supplier of the gels (Invitrogen/Life Technologies). A schematic of the method used is provided in Figure 1. After 24 hours, cells were rinsed with 10 mM Tris, pH 7.4, harvested by pipetting using a large bore tip in 50 mM Bis-Tris, 16 mM HCl, 50 mM NaCl, and centrifuged at 3000×g for 2 minutes. The cell pellet was resuspended in 240 µl the above buffer plus 1% Digitonin (Sigma-Aldrich: St. Louis, MO) and mixed on a Nutator for 30 min at RT. The lysed cells were pelleted at 3000×g for 2 minutes. The supernatant (S1) was centrifuged in a Beckman Airfuge for 5 min. This supernatant (S2) was mixed with 4x Sample NativePAGE sample buffer and G-250 sample additive and run on 3-12% Bis-Tris gel with Dark Blue Cathode buffer at 150 v for 45 min, with Light Blue Cathode Buffer at 150v for 15 min and with Light Blue Cathode buffer at 250 v for 1 hour. The protein was transferred to PVDF and fixed on the membrane either by air drying or incubation in 8% acetic acid for 15 min, followed by rinsing in water. Membranes were washed in methanol to remove Coomassie Blue and then water before immunoblotting. Note that we observed that when fixation was omitted, then we generally lost all signal on immunoblots. In some experiments (as noted in the Figure legends), the S1 fraction was analyzed directly by BNGE and immunoblotting. Because of the nature of the preparations, the samples were equalized volumetrically, using equal volumes of S1 or S2 supernatant after starting from a 60 mm dish of cells. In analysis of P2 pellet fractions, 1/10 of the sample was loaded.

## Results

To study the behavior of WT and mutant SOD1 in BNGE, we transiently transfected HEK293FT cells with expression plasmids for WT, A4V, G37R, G85R, G93A, and L126Z mutant SOD1. These mutants display a spectrum characteristics. For example, the A4V mutant is associated with rapidly progressing disease and shows a very high propensity to aggregate into detergent insoluble structures in this cell model [20], [26]. The G37R mutant is associated with a more slowly progressing form of fALS and displays a lower propensity to aggregate in the HEK293 cell model [20], [26]. The G85R and G93A mutants are associated with rapidly progressing disease and high propensity to aggregate [20] and have been well studied in mice. The L126Z mutant represents an example of a C-terminal truncation mutant that is turned over relatively quickly and shows a high propensity to aggregate [13].

**Figure 2:**
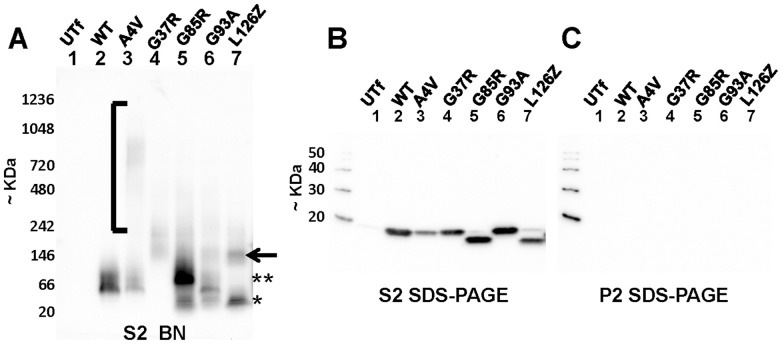
Immunoblots of BN-gels analyzing the electrophoretic mobility of WT and mutant SOD1. As described in Methods and diagrammed in Fig. 1, HEK293FT cells were transiently transfected with vectors to express WT or mutant SOD1. 24 hours post-transfection, the cells were lysed in digitonin and then soluble fractions were subjected to high speed centrifugation to generate an S2 soluble fraction and a P2 pellet fraction. (**A**) 16 µl of S2 soluble fraction was analyzed by BNGE and immunoblotting with hSOD1 antibody (which recognizes aa 24–36 of human SOD1 [32]). In this experiment, the PDVF membrane was air dried to fix proteins to the membrane. (**B and C**) To estimate loading and assess the level of large aggregates in the S1 fractions, 16 µl of the soluble fraction and 1/10 of total P2 fraction (roughly equivalent volumetrically to what is analyzed for the S2 fraction) were analyzed by SDS-PAGE and immunoblotting with the hSOD1 antibody. The membranes for each of the SDS-PAGE blots were incubated with primary and secondary antibodies in parallel (antibody dilutions prepared from the same stock and portioned out from a single mixture) and imaged simultaneously. The images shown are representative of at least 3 independent replications.

Relative to cells expressing WT-hSOD1 (Fig. 2, lane 2), cells expressing the mutant forms of hSOD1 all produced a faint smear of high molecular weight immunoreactivity that migrated between 200,000 kDa and 1.2 mDa (Fig. 2, lanes 3–8). In general, the A4V and G93A mutants most consistently produced immunoreactive high molecular weight smears. The G37R mutant produced a reactive entity that typically migrated slower than all other mutants, but in prior study of this mutant in native PAGE we have observed that the charge shifting mutation at this position has a major effect on electrophoretic mobility [7]. In cells expressing the G85R variant, there as an intense band of reactivity that migrated slightly slower than WT-hSOD1 in addition to the faint smear of high molecular weight reactivity (Fig. 2, compare lanes 1 and 5, double asterisks). The intensity of reactivity to high molecular weight entities formed by the G93A mutant varied considerably; in the example shown here the amount was unimpressive, but examples with more intense signals are provided below. The G93A and L126Z variants were unique in that lysates from cells expressing these variants routinely contained immunoreactive entities in a size range of ∼146 kDa that could, estimated from size alone, represent assemblies with a more homogenous composition (∼10 molecules). For most mutants, the relative intensity of reactivity to higher molecular weight entities was generally weak, but quantifying the relative amount of multimeric to monomeric forms of mutant protein in these gels is problematic because the mulitmeric reactivity was spread over a large area of the gel.

For the A4V, G85R, and G93A mutants, we observed immunoreactive entities that co-migrated with what was observed in cells expressing WT-hSOD1 (Fig. 2, compare lane 2 to lanes 3, 5, 6, and 7). The slower mobility of the reactivity for the G37R variant that we would predict to be monomeric precursor (based on abundance alone) maybe be due to the charge shift in the mutation (Fig. 2, lane 4). The G85R variant, which possess the same type of charge shifting mutation, does not produce the same effect (Fig. 2, lane 5). However, it is well established that the G85R variant exhibits aberrant electrophoretic mobility in SDS-PAGE [36] and we noted that the G85R variant produced a faster migrating entity on the BN-gels (Fig. 2, lane 5, asterisk). In cells expressing the G85R variant we observed a strongly reactive entity that migrated slower than WT-hSOD1 (2 asterisks) and could be an assembly of SOD1-G85R that is larger than a homodimer. For the L126Z mutant, we expect that the monomeric form of the protein should migrate more rapidly and we observe a faster migrating band for this mutant (Fig. 2, lane 8, asterisk). In general, it appeared that for the A4V, G85R, and G93A mutants a large percentage of the protein present in these cell lysates migrated to a size that could be interpreted as either monomeric or homodimeric.

To demonstrate that most of the proteins released from the cells into aqueous media by digitonin were indeed soluble, we centrifuged the samples at 100,000×g for 10 minutes in a Airfuge. A portion of this soluble material, which as analyzed by BNGE in panel A of Figure 2, was also analyzed by SDS-PAGE (Fig. 2B) along with the insoluble material (Fig. 2C). Very little of the material released into the aqueous media by digitonin was sedimented at 100,000×g, a finding consistent with our previous observations in treating cells with saponin or digitonin [30]. The SDS-PAGE gels, which focus all of the protein into a single band, provide a more clear indication of the levels of each mutant in the digitonin lysates (Fig. 2B). With the exception of the L126 mutant, which is known to turnover rapidly [13], the levels of all other expressed SOD1 variants were similar. Additionally, these data indicate that the vast majority of the SOD1 in the S2 fraction was not covalently modified in a manner that could produce high molecular weight smears (e.g. not ubiquitinated).

**Figure 3:**
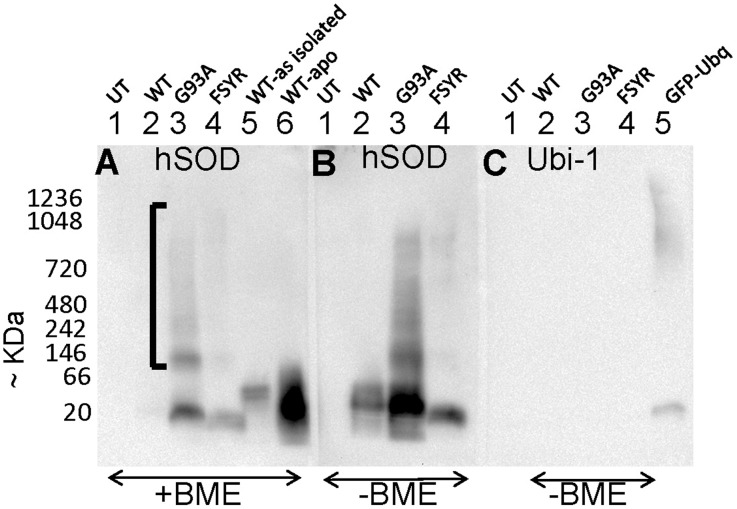
Disulfide crosslinking of mutant SOD1 contributes to electrophoretic heterogeneity. HEK293FT cells were transiently transfected with expression vectors for WT and mutant SOD1. After 24 hours, soluble extracts of the cells were prepared by lysis in digitonin, analyzed by BNGE, and immunoblotted with the antibody identified on the figure. (**A**) A portion of the cell lysate was treated with 2-mercaptoethanol (final concentration 1%) before electrophoresis. Gels containing samples treated with 2-mercaptoethanol (BME) were transferred separately from gels containing untreated samples. (**B and C**) A portion of the cell lysate was analyzed without BME treatment and immunoblotted with either antibody to SOD1 or antibody to ubiquitin. After transfer, the membranes were air dried to fix proteins to the membrane and then incubated with primary and secondary antibodies in parallel, with all three membranes imaged simultaneously. Treatment with 2-mercaptoethanol reduced amount of, but did not eliminate, slowly migrating G93A SOD1. A mutant form of SOD1 lacking all 4 cysteines produced low levels of slowly migrating forms of SOD1 immunoreactivity than the G93A mutant. The images shown are representative of at least 3 independent replications.

In prior studies we have determined that aberrant intermolecular disulfide cross links between SOD1 molecules can occur as the protein assembles into aggregates, but that such crosslinks play a limited role [27]. To determine whether disulfide bonding might be involved in the formation of the high molecular weight entities seen in immunoblots of BNGE, we performed 2 types of analyses. First, we electrophoresed the samples with 2-mercaptoethanol to reduce any disulfide bonds that might be present (Fig. 3A), and second we examined the behavior of an experimental mutant, termed SOD1-FSYR, in which all 4 cysteine residues have been mutated to Phe, Ser, Tyr, and Arg, respectively (see Methods and [27]). The SOD1-FSYR mutant has been shown to retain the capacity to form detergent insoluble complexes [27], and when fused to yellow fluorescent protein (YFP) to from large inclusion-like structures [37]. Including 2-mercaptoethanol in the loading buffer reduced, but did not eliminate, the higher molecular weight entities in cell lysates containing the SOD1-G93A variant (Fig. 3A, lane 3). In this gel, we electrophoresed preparations of purified hSOD1 that were either “as isolated” from yeast engineered to produce the human protein, or treated to remove Cu and reduce disulfide bonds (WT-apo) (Fig. 3A, lanes 5 and 6)[38]. By comparing the electrophoretic profile of the various samples, we could determine that the fastest migrating entity in cells expressing the G93A variant co-migrated with the WT-apo protein (Fig. 3A, compares lanes 3 and 6). In previous study, we have determined that most of the SOD1-G93A over-expressed in HEK293FT cells fails to acquire a normal intramolecular disulfide bond [38] and thus the migration pattern observed here is consistent with previous behaviors of this mutant in this cell culture system. As compared to the G93A variant, the lysates of cells expressing the experimental FSYR variant contained much less, but still detectable, levels of high molecular weight entities (Fig. 3A, lane 4). For comparison, we provide an immunoblot of the same cell lysates that were separated by BNGE in the absence of 2-mercaptoethanol and processed in parallel to panel A (exposure times equivalent). This comparison revealed that in general, including 2-mercapoethanol in the loading buffer, by some means, led to reduced immunoreactivity for all SOD1 entities in these cell lysates (Fig. 3, compare panels A and B).

In lysates of cells expressing the SOD1-FSYR variant, we observed very low, but still detectable, levels high-molecular-weight immunoreactive entities (Fig. 3). Because of the inherent variability with which we detect high molecular weight entities in these cells, it is difficult to definitively conclude that the low level of such entities in lysates of cells expressing the SOD1-FSYR variant is an indication of the role of disulfide bonding the formation of slowing migrating entities. It is possible that disulfide bonding occurs and helps to stabilize these entities, but is not the sole means by which high molecular weight entities can form.

Immunoblots of SDS-PAGE gels of SOD1 proteins released into lysates by digitonin indicated that the vast majority of the protein lacked covalent modifications (see Fig. 2B). To confirm that the high molecular weight smear of SOD1 immunoreactivity we observed in BN-gels was not due to ubiquitination of SOD1 protein, we immunoblotted cell lysates with an antibody to ubiquitin (Fig. 3C). No reactivity to this antibody was detected in lysates of cells expressing either WT or mutant SOD1. As a control for the ubiquitin antibody, we included a lysate from cells transfected with a vector that expresses a fusion protein of green fluorescent protein (GFP) and ubiquitin (Fig. 3C, lane 5). With an exposure equivalent to the exposure for panels A and B, we could detect the GFP ubiquitin protein, with no indication of reactivity to anything in the lysates of cells expressing the SOD1 proteins. Thus, the immunoblots with ubiquitin along with the immunoblots of the same fractions analyzed by SDS-PAGE (see Fig. 2B), indicate that the high molecular weight smear of mutant SOD1 seen in BNGE is not caused by polyubiquitination of the protein in these cells.

**Figure 4:**
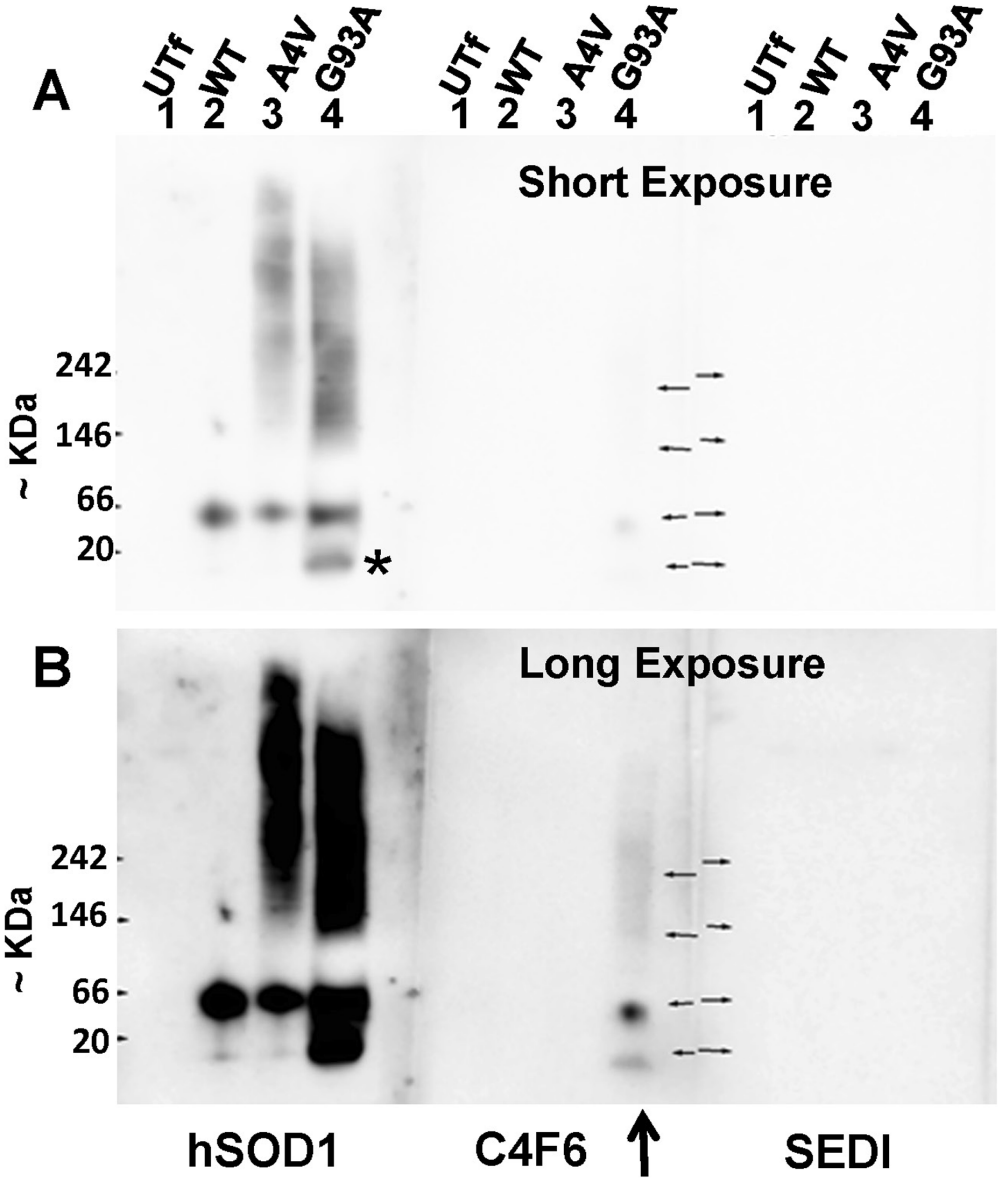
Slowly migrating forms of SOD1 in BN-gels do not react with C4F6 or SEDI antibody. Immunoblots of BN-gels containing the S1 fraction from lysates of cells expressing WT, A4V, and G93A SOD1 (see Methods) were probed with the hSOD1, SEDI, and C4F6 antibodies. Membranes were incubated in acetic acid to fix the proteins to the membrane and then incubated with antibodies as described in Methods. All membranes were developed in parallel with equivalent exposure times, both short and long exposures are provided to allow visualization of weak reactivity of the G93A variant with the C4F6 antibody. No reactivity to the A4V variant was detected with the C4F6 or SEDI antibody. The images shown are representative of at least 3 independent replications.

To determine whether the high molecular weight smears of SOD1 immunoreactivity are composed of misfolded SOD1, we probed immunoblots of BNGE gels with two antibodies that have shown specificity for misfolded SOD1. One of these antibodies, termed C4F6, is a monoclonal antibody that was raised against the apo form of G93A human SOD1 [33]. This antibody can immunoprecipitate the G93A protein as well as human SOD1 encoding other fALS mutations, but cannot immunoprecipitate WT-hSOD1 [33]. The other antibody, termed SEDI, is a rabbit polyclonal antibody that was raised against a peptide corresponding to amino acids 145–151 in hSOD1, located at the C-terminus of the protein, and which recognizes monomeric forms of hSOD1[34]. To assess relative reactivity, digitonin lysates of cells were aliquoted into 3 equal portions and analyzed by BNGE before immunoblotting (Fig. 4). In this experiment, the PVDF membranes were incubated in 8% acetic acid to fix the proteins to the membrane rather than air drying because we reasoned that air drying might denature the proteins. Immunoblots probed with the hSOD1 antibody revealed the SOD1 protein in the cell lysate. Faint reactivity to C4F6 was seen in cells expressing the G93A protein, with no particular specificity to higher molecular weight entities, but no reactivity was present in lysates of cells expressing A4V-hSOD1. No lysate of any cell contained entities reactive to SEDI antibody (Fig. 4).

## Discussion

In the present study, we have investigated the utility of using blue native gel electrophoresis as a means to detect soluble multimeric forms of mutant SOD1 in cell lysates. Our data show that in cells that over-express mutant SOD1 a sub-fraction of the protein shows electrophoretic migration patterns in BN-gels that are consistent with proteins that have assembled into multimeric structures. We find that intermolecular disulfide bonds may be involved in the formation of such structures, but are not likely to be the sole force stabilizing such structures. Although we observed C4F6 reactivity to SOD1-G93A, there was no remarkable binding of the C4F6 or SEDI antibodies, which show specificity to non-natively folded SOD1 [30], to high molecular weight entities of SOD1-A4V in immunoblots of BNGE. Overall, the data show that lysates of cells that over-express mutant SOD1 contain highly soluble forms of these proteins that are not covalently modified and yet show electrophoretic mobility in BN-gels that would be expected for multimeric assemblies.

Several prior studies have used size exclusion chromatography as a means to separate small mulitmeric assemblies of mutant SOD1 from larger aggregates, and from normal monomeric or dimeric forms of the protein [21]–[24]. In general, these studies have reported that, when detected, multimeric forms of mutant SOD1 are generally of low abundance. High molecular weight complexes of SOD1, in relatively low abundance, have also been detected in transiently transfected NSC34 cells by chemical cross-linking and SDS-PAGE analysis [39]. BNGE analysis offers a relatively less labor intensive approach to detecting soluble assemblies of protein of a size range predicted for multimeric proteins. To varying degrees, our data in BN-gels show that mutant SOD1 shows electrophoretic migration patterns in these gels that is consistent with multimeric structures. Whether such structures are homopolymeric assemblies of mutant SOD1 or heteromeric conglomerations of SOD1 with various other proteins is unknown.

As mentioned in the Introduction, the over-expression system we have used here would favor homopolymeric assemblies because the molar ratio of SOD1 to any preferred binding partner in the subset of cells expressing the mutant protein would be far higher than normal. It is possible that there is some abundant cellular protein that binds SOD1 and produces the electrophoretic migration patterns observed. Numerous SOD1 binding proteins have been described in the literature and among those described there are abundant proteins such as Hsp70 [18], [22], [40]–[43], Hsc70 [22], [42], [43], and the cytoplasmic dynein complex [44] (for a summary of other findings also see [45]). We would not expect the binding of a single protein to mutant SOD1 to produce the high molecular weight smears seen with the A4V or G93A proteins; such smears suggest either very heterogeneous complexes or homogeneous oligomeric structures of very heterogeneous size. Because of the nature of the system we have used to produce the proteins we favor that latter explanation but acknowledge that it is possible other proteins involved in the generation of the soluble non-native molecular weight forms of SOD1 observed in these BN-gels.

The fact that the mutant SOD1 in these cell lysates does not react with the C4F6 or SEDI antibodies does not provide corroborating evidence that the entities visualized with the hSOD1 antibody are multimeric assemblies of misfolded protein. We did observe some reactivity of G93A protein with the C4F6 antibody in immunoblots of BN-gels, but the G93A protein is the antigen used to produce C4F6 and the reactivity seen by this antibody for this protein could be the result of specific binding to the G93A protein, regardless of conformation [46]. The C4F6 antibody has been shown to be able to specifically react with mutant SOD1 proteins other than G93A [30], [33] and to WT protein that has been oxidatively modified [47] with such reactivity interpreted as evidence that the antibody recognizes a specific misfolded conformation. In a recent study, we observed that C4F6 could immunostain HEK293FT cells that over-express the A4V mutant and that most of these reactivity was released from cells by treatment with saponin or digitonin [30]. Thus we anticipated that immunoblotting of BN-gels would reveal the relative size of A4V protein within these cells that reacts with this antibody. The lack of reactivity of C4F6 to any form of the A4V mutant in these gels may suggest that most of the A4V protein in these cell lysates is not in a misfolded conformation that enables C4F6 binding. However, at present, we do not know whether the lack of reactivity indicates that there is so little C4F6 reactive A4V protein in these cells that it cannot be detected, or whether the epitope was lost in the process of BNGE and fixation to PVDF membrane. The lack of immunoreactivity to SEDI antibody to immunoblots of BN-gels similarly does not provide corroborating evidence that the high molecular weight entities in lysates of cells expressing mutant SOD1 are homopolymeric assemblies of misfolded protein. We note that homopolymeric assemblies of apo forms of SOD1 have been observed in crystal structures of mutant SOD1 [48], [49] and thus is it possible that we have detected oligomers formed by SOD1 proteins that are non-native but not necessarily misfolded.

In summary, we present evidence that cells that over-express mutant SOD1 can contain forms of SOD1 that migrate in BNGE in a manner expected for an multimeric assembly. Whether these more slowly migrating forms of SOD1 immunoreactivity we observed are homopolymers or heteropolymers is presently unknown. We were unable to observe reactivity of the A4V mutant with either the C4F6 or the SEDI antibody on these BN-gels. Whether this outcome reflects a feature of the protein in these gels or a consequence of the way the protein was presented to the antibody is uncertain and very difficult to assess. Taken on face value, our data from immunoblots of BN-gels provides evidence to corroborate findings from size exclusion chromatography [21]-[24], which suggests that fALS mutants of SOD1 can assemble into soluble multimeric structures.
